# Characteristics of colorectal cancer and use of colonoscopy before colorectal cancer diagnosis among individuals with inflammatory bowel disease: A population-based study

**DOI:** 10.1371/journal.pone.0272158

**Published:** 2022-08-01

**Authors:** Tawnya M. Hansen, Zoann Nugent, Charles N. Bernstein, N. Jewel Samadder, Sanjay K. Murthy, Harminder Singh

**Affiliations:** 1 Rady Faculty of Health Sciences, IBD Clinical and Research Center and Department of Internal Medicine, Max Rady College of Medicine, University of Manitoba, Winnipeg, Canada; 2 Division of Gastroenterology, Department of Medicine, Mayo Clinic, Scottsdale, Arizona, United States of America; 3 Department of Medicine, Division of Gastroenterology, The Ottawa Hospital IBD Centre, Ottawa, Ontario, Canada; School of Digestive & Liver Diseases, Institute of Post Graduate Medical Education & Research, INDIA

## Abstract

**Introduction:**

There are limited recent data on the characteristics of inflammatory bowel disease (IBD)-associated colorectal cancer (CRC) and the use of colonoscopy prior to CRC diagnosis among persons with IBD. We analyzed IBD-CRC characteristics, survival after IBD-CRC diagnosis and the use of colonoscopy prior to IBD-CRC diagnosis over time.

**Methods:**

We identified individuals with and without IBD from the University of Manitoba IBD Epidemiology Database and CRC from linkage to the Manitoba Cancer Registry. We compared characteristics of IBD-CRC and sporadic-CRC using logistic regression and survival after CRC diagnosis using Cox regression analysis. We assessed rate and predictors of colonoscopy use 5 years to 6 months prior to IBD-CRC.

**Results:**

1,262 individuals with CRC were included (212 IBD-CRC). IBD was associated with an increased risk of death after CRC diagnosis in 2004–2011 (HR 1.89; 95% CI 1.25–2.88) but not in 2012–2017 (HR = 1.002; CI 0.50–2.03). In the 5 years to 6 months prior to IBD-CRC (1989–2018), 51% underwent colonoscopy, which was very similar to IBD without CRC and contrasted to 9% among sporadic CRCs. Exposure to colonoscopy pre IBD-CRC remained stable through the study period (1989–2002 OR = 1.25; CI 0.77–2.01; 2003–2011 OR = 1.21; CI 0.56–1.70; reference 2012–2018). Exposure to colonoscopy pre-IBD-CRC was not associated with improved post-CRC survival.

**Conclusion:**

The risk of death following CRC diagnosis is not impacted by a diagnosis of IBD in recent years. There is a very high proportion of post colonoscopy CRC among IBD-CRC, which has not changed over the years and needs detailed root-cause analysis and interventions.

## Background

Colorectal cancer (CRC) continues to be the second most commonly diagnosed invasive cancer in North America [1]. Approximately 1–2% of CRC cases are inflammatory bowel disease (IBD)-associated and 10–15% of individuals with IBD will die from IBD-associated CRC (IBD-CRC) [2,3]. The presence of inflammation leads to mutational sequences, promoting the development of CRC, that do not follow the polyp-dysplasia-cancer pathway [4]. As a result, persons with IBD are diagnosed approximately 7 years earlier than persons with non-IBD associated CRC/sporadic CRC (sCRC) [5]. Using a national French registry, Peyrin-Biroulet *et al*. reported mean ages at CRC diagnosis of 56.9 years for individuals with IBD-CRC versus 70.9 years for those with sCRC between 1976–2008 (p<0.001) [5].

Several epidemiologic studies have reported worse IBD-CRC survival rates than for sCRCs but have not controlled for colonic cancer site [6–8] and only one prior study has controlled for stage [9]. Stratifying outcomes by site of CRC could be important since right sided CRCs (more commonly reported in Crohn’s disease [CD]) are associated with lower survival when compared to left sided CRCs [10]. Without adjusting for influential covariates, it can be difficult to ascertain whether survival from IBD-CRC is lower than the general population or whether these potential differences are secondary to comorbidities, tumor stage, or tumor site. Accurate assessments of survival are needed since differential survival after cancer diagnosis could direct different treatments and/or follow up after cancer diagnosis.

Surveillance with regular colonoscopies has been the cornerstone of the strategies to prevent CRC morbidity and mortality among those with IBD. Guidelines recommend colonoscopy at 3–5 year (or shorter) intervals for most individuals with longstanding IBD [11].

Much of the data on IBD-CRC predates the introduction of highly effective biological therapy for IBD and advances in diagnostic and therapeutic tests and strategies. Therefore, in this report, we aimed to assess if the characteristics of IBD-CRC, survival after IBD-CRC diagnosis, and use of colonoscopy pre-IBD-CRC diagnosis have changed over time compared to matched controls without IBD across the last two decades.

## Methods

### Data sources

Manitoba is a central Canadian province with a relatively stable population of approximately 1.3 million [12]. Manitoba Health is the single-payer health insurance provider in the province. All Manitobans have been assigned a unique personal health identification number (PHIN) since 1984, which can be used to track contact with the health care system. Manitoba Health maintains several administrative databases to monitor services delivered, including since 1984, hospitalization, discharge summaries, outpatient physician claims, and since 1995, prescription drug dispensation. A population registry is maintained to identify all residents of Manitoba with health insurance coverage and is updated with information on migration in and out of the province, births, and deaths. The University of Manitoba IBD Epidemiology Database (UMIBDED) was derived from this administrative dataset. The UMIBDED was established in 1995 and identifies all IBD cases using a validated case definition [13]. UMIBED data from 1984 to 2018 were used in the current study. Persons diagnosed with IBD were matched 1:10 with controls without IBD based on year of birth, sex, and the first three characters of the postal code.

Using Manitoba’s population-based Manitoba Cancer Registry (MCR), individuals diagnosed with CRC between 1956 and 2018 were identified in the current study. The MCR records the stage at diagnosis for all cancers 2004 (available to 2017 for the current study). The MCR has been consistently shown to be of very high quality including high levels of completeness and histological verification [14]. The UMIBDED data set includes about 10% of the CRC cases in the province.

### Study measures

Individuals with and without IBD were identified from the UMIBDED and those with CRC from the MCR. An individual with IBD was identified as a thiopurine user or 5ASA user if they had at least 2 dispensations of the respective medications and anti-TNF user if dispensed one prescription over the time period of follow-up included in the analysis. Only outpatient medication dispensations were included in this study as in-hospital pharmacy data are not accessible in the MH data.

The Socioeconomic Factor Index (SEFI) was used to assess socioeconomic status. SEFI utilizes neighbourhood level social determinants of health such as unemployment rate and age dependency ratio [15]. The SEFI has been validated in Manitoba and has been used in other IBD-related publications [15,16].

Comorbidity burden was determined from ambulatory care and hospital admission diagnoses in 3–15 months prior to index CRC using the Charlson Comorbidity Index (CCI) score.

The American Joint Committee on Cancer 6^th^ edition (AJCC6) classification system was used for cancer staging [17]. The data extracted from the MCR to conduct comparisons on CRC characteristics include: demographics (age at CRC diagnosis), site of CRC (proximal colon: cecum to splenic flexure, distal colon: descending/sigmoid colon, and rectal/rectosigmoid), histology, stage and grade.

### Statistical analyses

The baseline characteristics of persons diagnosed with CRC were compared using chi square and Fisher’s exact tests. Continuous variables (Age and SEFI) were analysed using Wilcoxon and Kruskal Wallis non-parametric rank tests. Multivariable logistic analysis was performed including factors significant at p≤0.10 in univariable analysis to determine differences between IBD-CRC and sCRC. Exposure to colonoscopy in the 5 years to 6 months before CRC diagnosis was determined for those with and without IBD. We compared characteristics of those who had colonoscopy before IBD-CRC versus those who did not.

Survival after CRC diagnosis was compared between persons with IBD-CRC and sCRC using proportional hazard regression models; covariates included age, sex, SEFI, site of CRC, year of CRC diagnosis and stage of CRC. Death from any cause was considered the endpoint. Hazard ratios (HR) with 95% confidence intervals (CI) were calculated. Similarly, predictors of survival after IBD-CRC diagnosis were assessed.

A nested case control analysis was performed to determine the association between CRC occurrence and colonoscopy 5 years to 6 months prior to CRC diagnosis. Individuals with IBD-CRC were matched (1:4) to individuals with IBD without CRC on the index date. Risk set sampling was used. Index date for this analysis was defined by the date of IBD-CRC diagnosis. Matching was based on age, sex, subtype of IBD (CD versus UC) and duration of IBD prior to CRC diagnosis. Conditional logistic regression analysis included covariates for SEFI, health care utilization, medication exposure, and colonoscopy exposure. Odds ratios (ORs) and 95% CIs were calculated. Because of the incidence density sampling, these ORs are unbiased estimates of incidence risk ratios.

Data were analysed in three time periods, each containing approximately one third of the IBD-CRCs: 1984–2002, 2003–2011 and 2012–2018. The data on stage of CRC were only available for the years 2004–2017. For analyses including previous colonoscopy history, CRCs diagnosed in the years 1989–2018 were included, to be able to evaluate colonoscopy in the 5 years pre-IBD-CRC.

In primary analysis, we used preceding 5 years to 6 months to define IBD-CRC as surveillance at 5-year intervals is recommended for some individuals with IBD. In a sensitivity analysis, we repeated the analysis for colonoscopy exposure 3 years to 6 months prior to IBD-CRC diagnosis.

Data management and analyses were completed using SAS version 9.4 (SAS Institute, Cary, NC). This study was approved by the University of Manitoba’s Health Research Ethics Board and MH’s Health Information and Privacy Committee. To protect patient confidentiality, the linkage in this study was performed, via scrambled PHINs using anonymous versions of the datasets. The ethics committee waived the need for consent from study subjects.

## Results

### Colorectal cancer among those with IBD compared to those without IBD

In total, 1,262 Manitobans diagnosed with CRC were included in the study, of whom 212 (16.8%) had IBD-CRC (58.5% UC; 41.5% CD) and the rest sCRC. 77 of the IBD-CRC occurred in individuals diagnosed with IBD prior to 1987 (defined as prevalent IBD cases for whom exact date of IBD diagnosis was not available) and had median duration of IBD at CRC diagnosis of more than 12 years (IQR 7–22). 135 IBD-CRC occurred among individuals diagnosed with IBD from 1987 and a median duration of IBD of 12 years (IQR 4–18 years) at CRC diagnosis. The median age at CRC diagnosis was approximately a decade younger among cases with IBD-CRC when compared to sCRC (Tables 1 and 2). MSI associated histology was more likely to be present in IBD-CRC when compared to sCRCs (21% versus 9%, p<0.001). There were no differences in stage or comorbidity. The proportion of individuals undergoing surgery, chemotherapy, and radiotherapy for their CRC did not differ between groups.

**Table 1:** Demographics of the study population with colorectal cancer stratified by inflammatory bowel disease diagnosis.

	IBD	No IBD	p-value	Crohn’s disease	Ulcerative colitis	p-value
N	212	1050		88	124	
% Male	59	54	0.17	48	68	0.0045
Median SEFI	-0.31	-0.25	0.93	-0.34	-0.31	0.37
N	211	1049		88	123	
Median age at CRC diagnosis (years)	59	69	<0.0001	59	59	0.35
% <50 year at CRC diagnosis	29	8	<0.0001	33	26	0.28
Era of CRC diagnosis						
1984–2002	77 (36%)	365 (35%)	0.87	29 (33%)	48 (39%)	0.28
2003–2011	73 (34%)	384 (37%)		30 (34%)	43 (35%)	
2012–2018	62 (29%)	301 (29%)		29 (33%)	33 (27%)	
Site of CRC (%)						
Proximal	43	40	0.0002	42	44	0.14
Distal	16	24		14	18	
Rectosigmoid/rectal	33	33		31	34	
Site unspecified	8	3		14	5	
Grade (%)						
	N = 157	N = 808		N = 67	N = 90	
1 or 2	78	85	0.043	76	80	0.56
3 or 4	22	15		24	20	
ACC6 Stage (%)+						
	N = 110	N = 607		N = 49	N = 61	
I	19	20	0.48	18	20	0.12
II	34	27		35	33	
III	27	31		18	34	
IV	20	22		29	13	
Histology (%)						
	N = 198	N = 1012		N = 82	N = 166	
Adenocarcinomas	76	86	<0.0001*	Suppressed**		0.22
Microsatellite Instability Associated	21	9				
Other	4	5				
Treatment						
Surgery (%)	87	83	0.26	82	90	0.099
Chemotherapy (%)	43	39	0.32	51	37	0.049
Radiation (%)	22	20	0.57	23	21	0.87
2+ prescriptions pre-CRC dx						
5ASA	72%			62%	79%	0.017
Thiopurines N (%)	173 (21)			76 (24)	97 (19)	0.45
Anti-TNF	135 (11)			59 (15)	76 (<10)	0.27
Years between IBD Dx and CRC Dx						
	N = 135			N = 62	N = 73	
Median	11.7			10.2	12.5	0.71
Interquartile range	4.5–17.6			3.1–18.0	5.0–17.6	

^+^ Stage data restricted to individuals diagnosed in the year 2004 and onward (IBD: 113, No IBD 614); medication usage analysis is restricted to those who were diagnosed in or after 1995 (5ASA, thiopurines) and 2003 (anti-TNF). Results are based on the whole sample numbers unless otherwise indicated.

*Chi-square used to ascertain p-value for MSI.

**Number less than 5 and excluded in order to protect privacy.

**Table 2:** Demographics of the study population with colorectal cancer stratified by era of diagnosis and IBD diagnosis*.

	1984–2002			2003–2011			2012–2018		
	IBD	No IBD	p-value	IBD	No IBD	p-value	IBD	No IBD	p-value
N	77	365		73	384		62	301	
% Male	60	54	0.45	58	54	0.61	61	54	0.33
SEFI Median	-0.45	-0.17	0.14	-0.02	-0.30	0.22	-0.37	-0.15	0.86
Age at CRC diagnosis (years)	56	69	<0.0001	61	69	<0.0001	60	68	0.001
Tumor Site (%)									
	N = 66	N = 344		N = 70	N = 377		N = 58	N = 298	
Proximal	44	39	0.75	47	40	0.21	50	45	0.31
Distal	23	26		17	27		12	21	
Rectosigmoid/ Rectal	33	34		36	33		38	34	
Grade (%)									
	N = 49	N = 271		N = 62	N = 304		N = 46	N = 233	
1/2	69	83	0.03	84	88	0.40	80	83	0.64
3/4	31	17		16	12		20	17	
% microsatellite unstable associated	32	13	0.0003	18	6	0.0036	8	8	0.79
Stage (%)									
				N = 60	N = 327		N = 50	N = 280	
I				25	21	0.16	12	19	0.57
II				33	26		34	27	
III				25	30		30	33	
IV				17	23		24	21	

*Wilcoxon rank test used for continuous variables.

Chi-square and Fisher’s exact tests used for discrete variables. Results are based on the whole sample numbers unless otherwise indicated.

In the multivariable analysis of the study population and including age, CRC site, grade and histology, individuals diagnosed with CRC before the age of 50 were 5 times more likely to be IBD-CRC than when the CRC was diagnosed after age 70 (OR: 5.11, 95% CI 3.03–8.65). For IBD among CRC 50–69 versus >70, OR was 1.79, 95% CI: 1.19–2.69. Those with distal CRC were less likely to be IBD-CRC than those with proximal CRCs (OR 0.47, 95% CI: 0.28–0.80).

### Colorectal cancer among those with UC compared to those with CD

When comparing CRC cases among persons diagnosed with CD and UC, persons with UC- CRC were more likely to be male (68% versus 48%, p = 0.0045). Persons with UC-CRC and CD-CRC were equally as likely to have seen a gastroenterologist in the 5 years to 6 months prior to CRC diagnosis (52% versus 49%, p = 0.77). UC-CRC versus CD-CRC did not differ in the median age of IBD-CRC diagnosis, colonic CRC site, or histology. There were no differences in exposure to thiopurines and anti-TNF therapies between persons with CD-CRC or UC-CRC. There was a higher proportion of 5ASA use among persons with UC-CRC. In a Cox regression model with 5ASA exposure a time dependent variable (exposure: 2 years after a prescription dispensation), and model co-variates age, sex, IBD subtype (UC vs. CD), there was no protective effect of 5ASA use on CRC risk (HR: 1.09; 95% CI: 0.79–1.49).

Longer disease duration was associated with a lower proportion of colonoscopy and gastroenterology visits in the 5 years-6 months before CRC-IBD diagnosis (Table 3). The proportion of individuals who underwent surgery, radiation, and chemotherapy did not vary by disease duration.

**Table 3:** Characteristics of IBD-CRC stratified by IBD disease duration prior to CRC diagnosis (CRC 2003–2018).

	Years between IBD diagnosis and CRC diagnosis			P*	Era of diagnosis		P**
	5 to <10 years	10 to <15 years	15+ years		2003–2011	2012–2018	
N	20	17	75	-	61	51	-
% with colonoscopy 5 years—6 months prior to IBD-CRC	65	47	45	0.15	51	47	0.71
% visited gastroenterologist 5 years—6 months prior to CRC-IBD	>70	65	55	0.05	62	59	0.85
Anti-TNF use before CRC %	<30	<35	9	0.45	<10	20	0.019
Tumor Site (%)							
Proximal	Suppressed	Suppressed	44	0.95	41	49	0.69
Distal			17		19	15	
Rectosigmoid/ Rectal			39		41	36	
Grade %							
1,2	62	>65	48	0.42	84	76	0.42
3,4	38	<35	52		16	24	
Stage %							
I/II	53	>65	48	0.41	59	44	0.21
III/IV	47	<35	52		41	56	
Treatment %							
Surgery	>70	>65	85	0.43	90	78	0.11
Chemotherapy	40	41	48	0.47	46	45	1
Radiation	<30	<35	23	0.42	20	22	0.82

*P-value from Mantel-Haenszel Exam;

**P-value from Fisher’s Exact and Chi-Square;

Exact values are not provided for the analysis where the number of individuals is less than 6 to protect patient privacy. Results are based on the whole sample unless otherwise indicated.

### Survival after colorectal cancer diagnosis

Because stage of disease is an important predictor of survival, analysis was restricted to the CRCs diagnosed in 2004 to 2017, for which staging was available. Overall, IBD was associated with increased mortality in the multivariable analysis with co-variates sex, age, colonic CRC site, year of CRC diagnosis, grade, histology and stage at CRC diagnosis (HR = 1.55; CI 1.09–2.20). In stratified analysis for CRC diagnosed 2004–11, IBD was associated with increased mortality (HR = 1.89; CI 1.25–2.88) but not among CRC diagnosed in 2012–2017 (HR = 1.003; CI 0.50–2.03). More specifically, one- (HR = 0.49, 95% CI: 0.14–1.72) and three-year (HR 0.62, 95% CI:0.26–1.44) survival rates did not differ between those diagnosed with and without IBD among individuals diagnosed with CRC in 2012–2017.

In the multivariable analysis limited to IBD-CRC, individuals with shorter duration IBD prior to IBD-CRC diagnosis had better survival after CRC diagnosis (<5 years: HR 0.36, 95% CI: 0.14–0.88; 5–10 years: HR 0.31, 95% CI: 0.12–0.78; 10–15 years HR 0.94, 95% CI 0.43–2.08 with duration 15+ years as reference). UC vs. CD diagnosis, age, sex, anti-TNF use, thiopurine use, 5-ASA use and being seen by a gastroenterologist in the 5 years to 6 months prior to CRC diagnosis did not impact survival among individuals with IBD-CRC. There was no effect of colonoscopy exposure in the 6 months to 5 years prior to IBD-CRC on survival after CRC diagnosis (HR 1.01, 95% CI 0.51–2.01). Among those with UC-CRC, diagnosis of UC in hospital (marker of disease severity) did not impact survival after IBD-CRC diagnosis.

### Colonoscopy exposure 5 years to 6 months prior to CRC diagnosis

**Fig 1:**
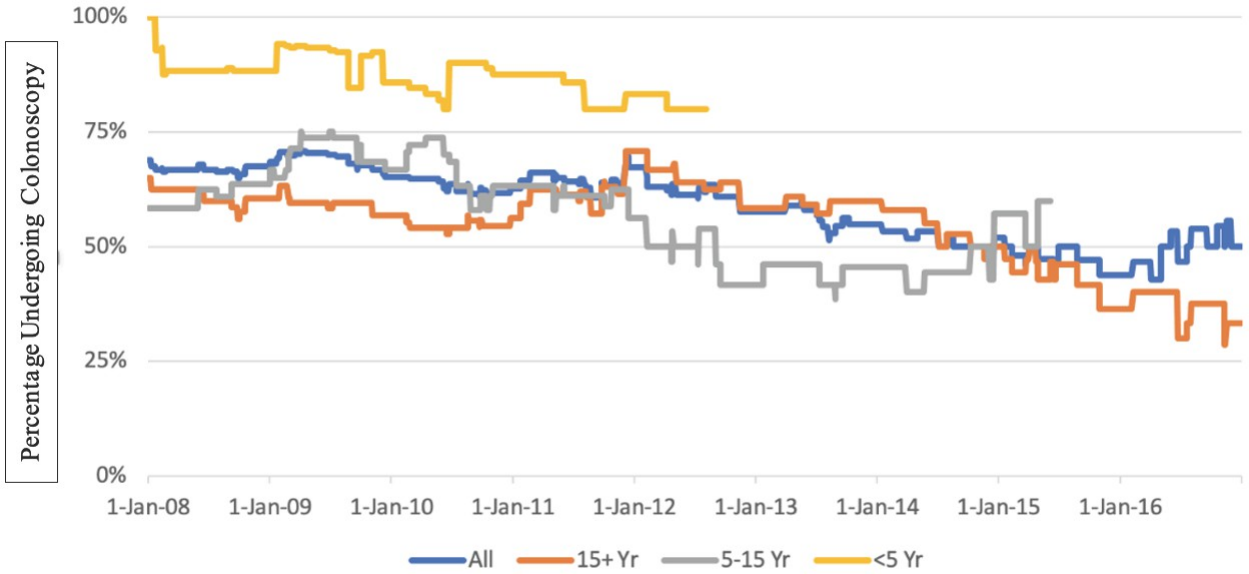
Colonoscopy use 5 years to 6 months prior to CRC diagnosis for those with IBD-CRC (censored 6 months prior to CRC diagnosis).

In the 5 years to 6 months prior to CRC diagnosis (1989–2018), 51% of individuals with IBD-CRC underwent a colonoscopy versus 9% of sCRCs (OR 6.86, 95% CI: 4.56–10.34). Exposure to colonoscopy in the 5 years to 6 months prior to IBD-CRC diagnosis was similar for those diagnosed with CRC in the latest two time periods (2003–2011: 37 of 68 = 54%; 2012–2018: 33 of 61 = 54%) The exposure to colonoscopy pre IBD-CRC diagnosis decreased slightly over time among those with long-standing IBD (> 15 years IBD duration) (Fig 1). Stage at time of CRC diagnosis did not vary between those who did have a colonoscopy and those who did not have a colonoscopy in the 5 years to 6 months prior to CRC diagnosis (Stage III/IV 46% vs 48%, p = 0.85) (Table 4).

**Table 4:** Colonoscopy use in the 5 years to 6 months prior to IBD-CRC diagnosed in 2003–2018*.

	Individuals who did not undergo colonoscopy (n = 59)	Individuals who underwent colonoscopy (n = 70)	P value
Age (%)			
<50	29	23	
50–75	44	59	0.83
75+	27	18	
Male (%)	56	63	0.47
Era			
2003–2011	53%	53%	1.00
2012–2018	47%	47%	
Gastroenterology Visit 5 years– 6 months prior to IBD-CRC diagnosis	44%	80%	<0.0001
• Persons with disease duration >8 years	41%	79%	0.0001
• Persons with disease duration >10 years	36%	81%	<0.0001
• Persons with disease duration >15 years	34%	79%	0.0002
Surgeon visit 6 months to– 5 years prior to IBD-CRC diagnosis	31%	60%	0.0013
• Persons with disease duration >8 years	31%	57%	0.0099
• Persons with disease duration >10 years	32%	62%	0.0061
• Persons with disease duration >15 years	29%	59%	0.018
Anti-TNF prior to IBD-CRC	10%	13%	0.78
Charlson Co-morbidity Index Score (%)			
0	71	57	0.17
1	14	23	
2+	15	20	
SEFI (Median)	-0.35	-0.11	0.65
UC	29 (39%)	45 (61%)	0.11
CD	30 (55%)	25 (45%)	
Duration of IBD prior to CRC			
Median (IQR)	16.7 (13.3–20.8)	9.6 (4.1–17.6)	0.0009
Colon Cancer Characteristics			
Stage I/II N (%)	25 (52%)	31 (54%)	0.85
Stage III/IV (%)	23 (48%)	26 (46%)	
% MSI associated histology	15%	11%	0.61
Proximal site	28 (50%)	29 (43%)	0.76
Distal site	8 (14%)	11 (16%)	
Rectosigmoid/rectum	20 (36%)	27 (40%)	
Follow up after CRC diagnosis (Median, IQR)	2.9 (0.5–6.9)	2.9 (1.5–6.2)	0.48
Colonoscopy exposure pre CRC (yes/no) and risk of death after CRC	Univariable: HR = 0.93 Multivariable; HR = 1.01	95% CI 0.56–1.54 95% CI 0.51–2.09	
5ASA (%)	71	84	0.088
Thiopurines (%)	29	21	0.41

*Wilcoxon rank test used for continuous variables.

Chi-square and Fisher’s exact tests used for discrete variables.

Exposure to colonoscopy in a 5-year time period for all individuals with longstanding IBD (> 15 years IBD duration, with or without CRC, increased from 50% to 59% from 2008 to 2018 (S1 Fig). When stratified by age, 68% of persons 50–69 had a colonoscopy in the preceding 5 years (S2 Fig).

In the multivariable model assessing predictors of use of colonoscopy in the 5 years to 6 months preceding IBD-CRC, those who saw a gastroenterologist in the 5 years to 6 months prior to IBD-CRC were almost 5 times more likely (Table 5) to have a colonoscopy compared to those who did not see a gastroenterologist. The other covariates of age at CRC diagnosis and era of CRC diagnosis had no significant effect.

**Table 5:** Multivariable logistic regression analysis assessing use of colonoscopy in the 5 years to 6 months prior to IBD-CRC.

	OR	95% CI
Gastroenterology visit 5 years to 6 months prior (yes versus no)	4.87	2.54–9.34
<50	0.96	0.42–2.19
50–69	1.48	0.71–3.08
70+	Reference	
1989–2002	1.21	0.54–2.69
2003–2011	0.97	0.47–2.01
2012–2018	Reference	

In the nested case control conditional logistic regression analysis among individuals with IBD with and without CRC, there was no association between use of colonoscopy and IBD-CRC. 55% of IBD-CRC versus 51% of IBD without CRC had one or more colonoscopy in the 5 years to 6 months prior to the index date (date of CRC) (OR = 1.15, 95% CI 0.79–1.66). Individuals with IBD-CRC had more frequent colonoscopies in this time frame (26% one colonoscopy, 17% two and 12% three or more) than individuals with IBD without CRC (32% one colonoscopy, 12% two and 7% three or more colonoscopies) (p = 0.028). Eighty five percent of 5 years to 6 months pre index date colonoscopies for IBD-CRC included a biopsy as did 84% of colonoscopies for IBD without CRC (p = 0.87).

### Colonoscopy exposure 3 years to 6 months prior to IBD-CRC diagnosis

About one-third of individuals underwent colonoscopy in the 6–36 months prior to IBD-CRC (1987–1999: 43%; 2000–2009: 34%; 2010–2018: 35%). For those diagnosed with IBD-CRC between 2003–2018, seeing a gastroenterologist was associated, with undergoing a colonoscopy prior to IBD-CRC diagnosis (S1 Table). Of the individuals who did not have a colonoscopy in the 3 years to 6 months prior to IBD-CRC diagnosis (2003–2018), 45% had disease duration of > 8 years and had been seen by a gastroenterologist. There were no significant differences in age, sex, era of diagnosis, medication use, stage, or site of CRC between those who did or did not undergo colonoscopy prior to IBD-CRC diagnosis. In the multivariable model also, age at CRC diagnosis and era of CRC diagnosis had no significant effect on use of colonoscopy in the 3 years to 6 months preceding IBD-CRC (S2 Table).

## Discussion

In this population-based study, a diagnosis of IBD was not associated with an increased risk of death after CRC diagnosis in the 2012–2017 era, and 51% of individuals diagnosed with IBD-CRC had post colonoscopy CRC (PCCRC), defined as CRC diagnosed within 5 years of a colonoscopy (and one-third within 3 years). Although persons with IBD-CRC, who saw a gastroenterologist before their CRC diagnosis were almost 5 times more likely to have a colonoscopy than those who did not see a gastroenterologist, exposure to colonoscopy pre- IBD-CRC did not confer a survival advantage after CRC diagnosis. Persons with IBD-CRC were younger and with more proximal CRC. We found a large proportion of individuals with longstanding IBD (> 8 years) did not have a colonoscopy in a 5-year time interval.

Previously, individuals with IBD-CRC were reported to have lower odds of survival following CRC diagnosis when compared to the general population [18]. In our study, there was no effect of IBD on survival after CRC diagnosis in the latest time period included in the study. It is possible, medical, and surgical improvement in CRC management over the years have eliminated the differential effect of IBD.

PCCRC has been estimated to account for 3.7% of CRCs diagnosed in the general population [19]. In comparison, Wintjens and colleagues reported that 45% of all CRCs in the Inflammatory Bowel Disease South Limburg Cohort (IBDSL) were preventable post-colonoscopy CRCs (PCCRCs) [20]. These cancers were classified as any CRC diagnosed within 5 years of a colonoscopy and the majority of these lesions were classified as true missed lesions. Other reasons for PCCRCs were incomplete resection, inadequate bowel examination, and inappropriate interval for surveillance. Accounting for variations in the definition of PCCRCs, it is estimated that 21–29% of CRCs detected in European IBD surveillance cohorts are due to missed lesions [20,21]. In the largest study to date, Stärngrim and colleagues used the Swedish National Patient Register to compare the rates of PCCRC among persons with IBD to those without IBD [22]. They reported high relative risks for PCCRC (CRC detected 6–36 months post-colonoscopy) among those with CD (3.82, 95% CI: 2.94–4.96) and UC (5.89, 95% CI 5.10–6.80), finding that the highest risks were among young persons with UC and rectal cancers among those with CD [22]. In our cohort, 51% of individuals developed post colonoscopy IBD-CRC and in nested case control analysis undergoing a single colonoscopy did not decrease the risk of IBD-CRC. Among those with IBD diagnosis more than 5 years prior to CRC diagnosis 46% underwent a colonoscopy in the 5 years to 6 months prior to IBD-CRC diagnosis. One possible reason for these findings is that a significant proportion of cancers or high-grade dysplastic lesions were missed. A lack of survival difference for those undergoing colonoscopy in the 5 years to 6 months prior to CRC diagnosis supports this. More attention to lesion identification during endoscopy training or increasing the time for surveillance colonoscopies is likely needed. Careful reflection is required to identify aspects of care that could be optimized to improve patient outcomes. It is also possible that some of the prior colonoscopies were truly negative. IBD-CRCs are thought to develop faster than sCRCs and do not follow the traditional adenoma-cancer sequence [23]. Irrespective of the etiology of PCCRC among those with IBD, these data highlight it is essential to develop interventions to decrease risk of post colonoscopy IBD-CRC.

Individuals with IBD undergo colonoscopy at a higher frequency than the general population and this has been suggested to be another possible explanation for the high percentage of PCCRCs in IBD-CRC [24]. Troelsen and colleagues conducted a cohort study to determine whether the high rates of PCCRCs in persons with IBD could be attributed to colonoscopy frequency [24]. In their study, they calculated the cumulative incidence proportions of PCCRC for persons with IBD (0.21%; 95% CI 0.17%-0.27%) and persons without IBD (0.37%, 95% CI 0.75%-1.2%). Yet, the 3-year PCCRC rate was higher among those with IBD compared to those without IBD (24.3% versus 7.5%). This calculation highlights that risk of PCCRC is low after any one colonoscopy, which itself should be reassuring to patients, but does not alter that colonoscopy does not seem to prevent a large proportion of future CRC diagnosis in IBD.

The results of this study should be considered in the context of strengths and weakness. This is a population-based study evaluating practices, outcomes and time trends over a long time period. Our data are therefore not limited by selection bias. However, we did not have access to clinical charts, IBD phenotype or disease activity. The study was limited in its ability to report on disease characteristics such as duration of disease. Indications for colonoscopy could not be determined from the administrative health care data. Data on colonoscopy surveillance should be considered in the context of an entire cohort of IBD, without stratification for intestinal extent of IBD or indications of colonoscopy. Lastly, colonoscopy quality indicators could not be accounted for thus, the cause for PCCRC could not be determined.

In conclusion, this study suggests short term survival after IBD-CRC diagnosis has improved recently. Longer follow up of this cohort will be required to determine if long term survival is also improved. Previous studies findings that CRC is increased in persons with IBD compared to matched controls and high risk of PCCRC among IBD-CRC further suggests that we must be vigilant and careful with surveying patients with IBD involving the colon for CRC. Importantly, since a large proportion of individuals with IBD-CRC had a colonoscopy within the preceding 6 months to 5 years of CRC, measures need to be developed to decrease these post colonoscopy IBD-CRCs.

## Supporting information

S1 Fig Colonoscopy use stratified by disease duration for all individuals with IBD, regardless of CRC status. (DOCX)

S2 Fig Colonoscopy use stratified by age for all individuals with IBD, regardless of CRC status. (DOCX)

S1 Table Colonoscopy use in the 3 years to 6 months prior to IBD-CRC diagnosed in 2003–2018*. (DOCX)

S2 Table Multivariable logistic regression analysis assessing use of colonoscopy in the 3 years to 6 months prior to IBD-CRC. (DOCX)
