# Supplementary material for: Characteristics of colorectal cancer and use of colonoscopy before colorectal cancer diagnosis among individuals with inflammatory bowel disease: A population-based study
Source: PLoS One. 2022 Aug 1;17(8):e0272158. doi: 10.1371/journal.pone.0272158 (PMC9342763; doi:10.1371/journal.pone.0272158)
Supplement: S2 Fig — (DOCX) [file pone.0272158.s002.docx]

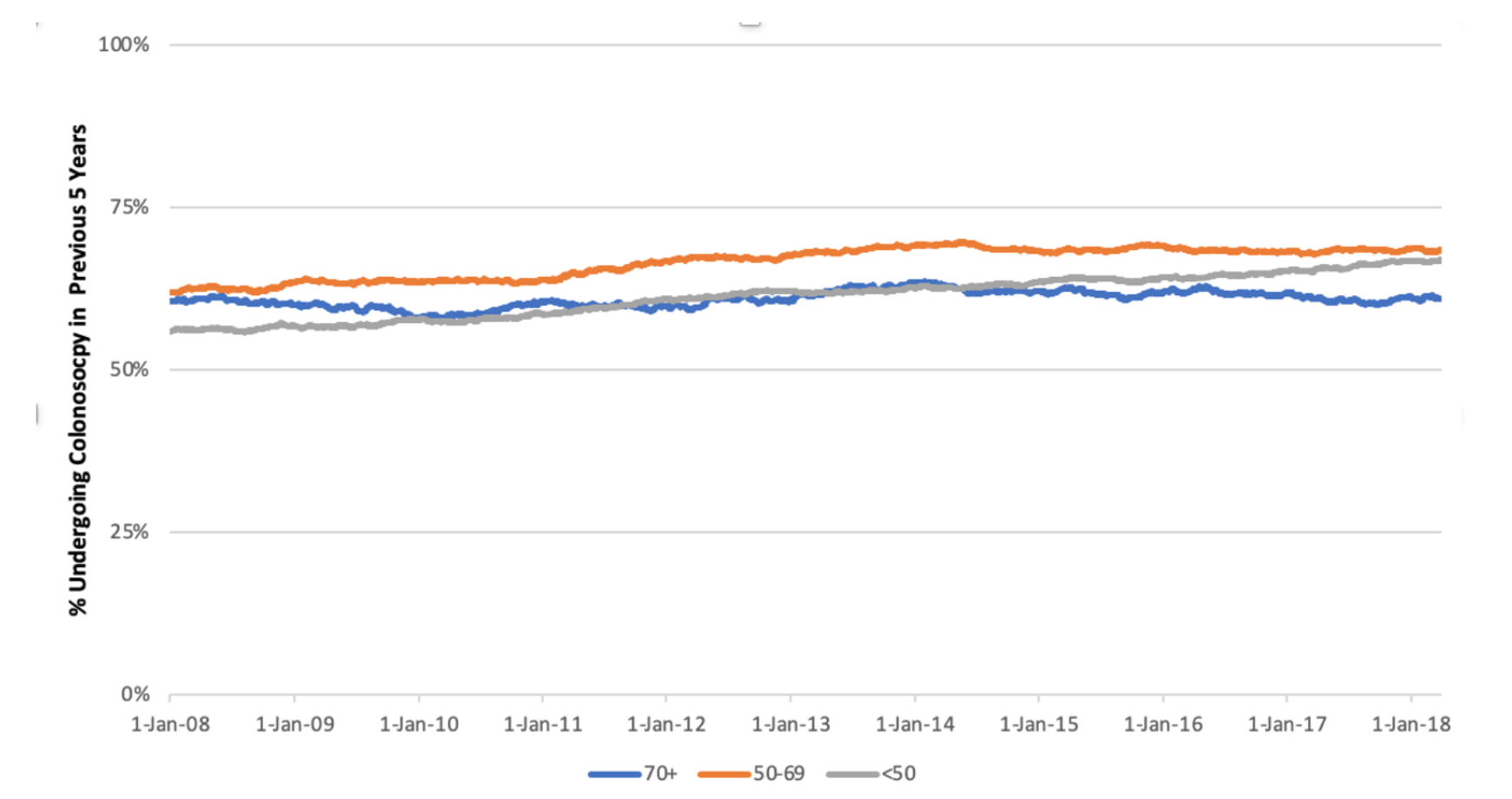


**Supplementary Figure B .** Colonoscopy use stratified by age for all individuals with IBD, regardless of CRC status
