# Supplementary material for: Characteristics of colorectal cancer and use of colonoscopy before colorectal cancer diagnosis among individuals with inflammatory bowel disease: A population-based study
Source: PLoS One. 2022 Aug 1;17(8):e0272158. doi: 10.1371/journal.pone.0272158 (PMC9342763; doi:10.1371/journal.pone.0272158)
Supplement: S1 Table — (DOCX) [file pone.0272158.s003.docx]

**Supplementary Table A: Colonoscopy use in the 3 years to 6 months prior to IBD-CRC diagnosed in 2003-2018***

|  | Individuals who did not undergo colonoscopy (n=78) | Individuals who underwent colonoscopy (n=51) | P value |
| --- | --- | --- | --- |
| Age % |  |  |  |
| <50 | 72 | 24 | 0.21 |
| 50-75 | 46 | 61 |  |
| 75+ | 27 | 16 |  |
| Male (%) | 56 | 65 | 0.37 |
| Era (%) |  |  |  |
| 2003-2011 | 51 | 55 | 0.72 |
| 2012-2018 | 49 | 45 |  |
|  |  |  |  |
| % with Gastroenterology Visit 3 years- 6 months prior to IBD-CRC diagnosis | 51 | 82 | 0.0004 |
| Persons with disease duration >8 years | 45 | 83 | 0.0003 |
| Persons with disease duration >10 years | 42 | 81 | 0.0004 |
| Persons with disease duration >15 years | 40 | 81 | 0.0006 |
|  |  |  |  |
| % with Surgeon Visit 3 years- 6 months prior to IBD-CRC diagnosis | 33 | 47 | 0.14 |
| Persons with disease duration >8 years | 33 | 46 | 0.28 |
| Persons with disease duration >10 years | 34 | 48 | 0.19 |
| Persons with disease duration >15 years | 29 | 44 | 0.21 |
|  |  |  |  |
| Anti-TNF prior to IBD-CRC (%) | 9 | 16 | 0.27 |
|  |  |  |  |
| Charlson Co-morbidity Index Score (%) |  |  |  |
| 0 | 69 | 55 | 0.14 |
| 1 | 15 | 24 |  |
| 2+ | 15 | 22 |  |
|  |  |  |  |
| SEFI (Median) | -0.39 | 0.32 | 0.13 |
| N | 77 | 51 |  |
|  |  |  |  |
| UC | 38 (69%) | 17 (31%) |  |
| CD | 40 (54%) | 34 (46%) |  |
| Duration of IBD prior to CRC |  |  |  |
| Median (IQR) (years) | 17.5 (10.2-23.0) | 15.3 (6.6-24.6 | 0.19 |
|  |  |  |  |
| Colon Cancer Characteristics |  |  |  |
| Stage I/II N (%) | 33 (52%) | 23(55%) | 0.84 |
| Stage III/IV (%) | 30 (48%) | 19 (45%) |  |
|  |  |  |  |
| % MSI associated histology | 17 | Suppressed | 0.19 |
|  |  |  |  |
| Proximal site | 32 (53%) | 18 (45%) | 0.23 |
| Distal site | 6 (10%) | 9 (23%) |  |
| Rectosigmoid/rectum | 22 (37%) | 13 (32%) |  |
|  |  |  |  |
| Follow up after CRC diagnosis (Median, IQR) | 2.9 (0.5-6.9) | 2.8 (1.1-6.2) | 0.63 |
| Colonoscopy exposure pre CRC (yes/no) and risk of death after CRC | Univariable: HR = 0.93 | 95% CI 0.56-1.56 |  |
|  | Multivariable; HR=0.77 | 95% CI 0.42-1.40 |  |
|  |  |  |  |
| 5ASA (%) | 53 | 59 | 0.59 |
| Thiopurines (%) | 12 | 17 | 0.60 |

*Wilcoxon rank test used for continuous variables. Chi-square and Fisher’s exact tests used for discrete variables. Results are based on the whole sample numbers unless otherwise indicated.
