# Supplementary material for: Characteristics of colorectal cancer and use of colonoscopy before colorectal cancer diagnosis among individuals with inflammatory bowel disease: A population-based study
Source: PLoS One. 2022 Aug 1;17(8):e0272158. doi: 10.1371/journal.pone.0272158 (PMC9342763; doi:10.1371/journal.pone.0272158)
Supplement: S2 Table — (DOCX) [file pone.0272158.s004.docx]

**Supplementary Table B. Multivariable logistic regression analysis assessing use of colonoscopy in the 3 years to 6 months prior to IBD-CRC**

|  | OR | 95% CI |
| --- | --- | --- |
| Gastroenterology visit 3 years to 6 months prior (yes versus no) | 4.70 | 2.45-9.02 |
| <50 | 1.23 | 0.52-2.92 |
| 50-69 | 1.75 | 0.81-3.77 |
| 70+ | Reference | |
| 1989-2002 | 1.31 | 0.59-2.94 |
| 2003-2011 | 1.00 | 0.47-2.13 |
| 2012-2018 | Reference | |
